# Two octaves spanning photoacoustic microscopy

**DOI:** 10.1038/s41598-022-14869-5

**Published:** 2022-06-22

**Authors:** Gianni Nteroli, Manoj K. Dasa, Giulia Messa, Stella Koutsikou, Magalie Bondu, Peter M. Moselund, Christos Markos, Ole Bang, Adrian Podoleanu, Adrian Bradu

**Affiliations:** 1 Applied Optics Group, University of Kent, Canterbury, UK; 2 DTU Fotonik, Technical University of Denmark, 2800 Kgs. Lyngby, Denmark; 3 Medway School of Pharmacy, University of Kent, Chatham, UK; 4 NKT Photonics A/S, Blokken 84, 3460 Birkerød, Denmark

**Keywords:** Applied optics, Photoacoustics, Optics and photonics, Microscopy

## Abstract

In this study, for the first time, a Photoacoustic Microscopy instrument driven by a single optical source operating over a wide spectral range (475–2400 nm), covering slightly more than two octaves is demonstrated. *Xenopus laevis* tadpoles were imaged in vivo using the whole spectral range of 2000 nm of a supercontinuum optical source, and a novel technique of mapping absorbers is also demonstrated, based on the supposition that only one chromophore contributes to the photoacoustic signal of each individual voxel in the 3D photoacoustic image. By using a narrow spectral window (of 25 nm bandwidth) within the broad spectrum of the supercontinuum source at a time, in vivo hyper-spectral Photoacoustic images of tadpoles are obtained. By post-processing pairs of images obtained using different spectral windows, maps of five endogenous contrast agents (hemoglobin, melanin, collagen, glucose and lipids) are produced.

## Introduction

Photoacoustic Microscopy (PAM) is a rapidly emerging, non-ionizing, cross-sectional imaging technique, which provides structural and functional volumetric information with micrometre resolution. Most importantly, it allows non invasive detection of chromophores, paving the road for applications in medical diagnosis and oncology, as well as in biology and neuroscience^1–8^.

Currently, PAM instruments operate over narrow spectral ranges where a small number of chromophores can be identified, hence their limited spectral capabilities. Such PAM instruments were developed for example either in the 532 nm^9,10^, 900 nm spectral bands^11,12^, or above 1440 nm^13^.

Producing simultaneously PAM maps of several biological chromophores, such as Hb, melanin, collagen, glucose, and lipids is a challenging task for the available instrumentation. Several research groups have already demonstrated PAM’s capability to produce oxygen saturation maps. For example, Liu et al measured simultaneuosly Hb concentration, oxygen saturation and blood flow speed via PAM, using 3 laser lines. For example, Liu et al. measured simultaneously Hb concentration, oxygen saturation and blood flow speed via PAM, using 3 laser lines^10^. This was achieved thanks to a single nanosecond pulsed laser operating at 532 nm and two stimulated Raman shifted pulses at 545 and 558 nm. By using a high numerical aperture acoustic lens, Li et al.^14^ performed in vivo oxygen saturation imaging with only 10 nJ per pulse, at 532 nm without the need of data averaging. Galanzha et al.^15^ employed in vivo PAM flow cytometry for label-free detection of melanin-bearing circulating tumor cells in patients with melanoma, using a pulsed laser operating at 1060 nm. On the other hand, supercontinuum (SC) optical sources provide a wide spectral range (typically 500–2400 nm), enabling multi-spectral PA imaging and spectroscopic PA sensing, despite exhibiting challenging low pulse energies. Shu et al.^16^ demonstrated a single SC source powered OCT/PAM instrument capable of multi-spectral microscopy (MS-PAM) in the visible (500–800 nm). Also, Buma et al.^17^ and Dasa et al.^13^ developed in-house SC sources to map lipids in the near infra-red (NIR). Table 1, presents a review of the instrumentation of PAM systems employed to target absorbers in biological tissues. This shows that, currently, the imaging instrument needs to be tailored to match the spectral characteristics of the chromophore of interest.

**Table 1:** Review of the instrumentation used for PAM imaging of hemoglobin (A), melanin (B), collagen (C), glucose (D) and lipids (E). *OS* optical source employed, *CWL* central wavelength of OS, *PRR* pulse repetition rate, *PLD* pulsed laser diode, *OPO* optical parametric oscillator, *SC* supercontinuum source, *YFL* Ytterbium fibre laser, *EPP* energy per pulse.

Reference	OS Tech.	CWL (nm)	PRR	Cost	Target	EPP (μJ)
^18^	PLD	405	1 kHz	Low	A	0.052
^11^	PLD	905	1 kHz	Low	A	3
^9^	YFL	532	2 MHz	High	A	0.1–5
^10^	YFL	532	4 kHz	High	A	0.8
^19^	Dye Laser	532	10 kHz	High	A	5
^20^	PLD	780, 1560	5–20 kHz	N/A	C	2–4
^21^	CW	488, 808	N/A	Low	A,B	N/A
^15^	N/A	1060	10 kHz	N/A	B	240
^22^	OPO	270–1400	1 kHz	High	A, E	N/A
^23^	SC	450–840	20 kHz	Low	A, B	N/A
^16^	Custom SC	500–900	5 kHz	Low	A, B	N/A
^17^	Custom SC	1210–1720	2 kHz	High	E	1–6
^13^	Custom SC	1440–1870	100 kHz	Low	E	0.197
^24^	Custom SC	1500–1900	30 kHz	Low	D	1
OS-PAM	SC	475–2400	20 kHz	Low	A, B, C, D, E	0.017–0.11

Several research groups have attempted to develop low-cost PAM imaging instruments. To this goal, pulsed laser diodes (PLD) operating in the visible and NIR region were employed. However, PLD based PAM instruments exhibit a low pulse repetition rate (PRR). The low PRR results in slow imaging rate and low pulse energies leads to higher averaging of the detected signals by hundreds of times^11–13,18,25–27^. Other approaches include frequency-domain (FD) systems. For example Kellnberger et al.^21^ used a CW laser emitting at 488 and 808 nm, whilst Tserevelakis et al.^28^ employed a CW laser (at 488 nm) with an acousto-optic modulator at 10 MHz. In contrast to PLD based PAMs and FD CW laser based systems, a more costly instrument, was reported by Allen et al.^9^. They set up an ultra-fast PAM system with a PRR of 2 MHz using a master oscillator power amplifier configuration, frequency-doubled to 532 nm. So far, optical parametric oscillators (OPO) are popular optical sources employed for PAM. Although they are widely tunable in wavelength, they are characterized by a low PRR^22^. Recently, mapping of lipids using a low cost customised SC source to deliver sufficiently high energy per pulse (EPP) in the NIR was reported by Dasa et al.

To our knowledge, an optical source which,

(i) has the ability to cover both VIS and NIR ranges
(ii) can deliver sufficient EPP to enable PAM imaging
(iii) provides sufficiently high PRR for fast imaging
(iv) is cost effective

has not been reported yet. A source that addresses all criteria (i) to (iv) listed above is a commercial low-cost SC source employed for this study, whose wide emission spectrum enables to target several endogenous chromophores, with absorption peaks spread over a spectral region of almost 2000 nm (from 475 to 2400 nm).

Using such a source, for the first time, a two Octaves Spanning Photoacoustic Microscopy (OS-PAM) instrument is demonstrated. The instrument is capable of identifying chromophores over a spectral range of nearly 2000 nm across the electromagnetic spectrum, and can produce 3D PAM maps of various chromophores (melanin, hemoglobin (Hb), collagen, glucose and lipids) in real-time. In addition, we created an advanced multimodal imaging instrument (OS-PAM/OCT) by integrating a high-resolution Optical Coherence Tomography (OCT) imaging channel. The wide bandwidth covered and the multimodality demonstrated recommend such instruments for future studies of early stage cancer detection.

## Theoretical background/distinguishing different contrast agents

**Figure 1:**
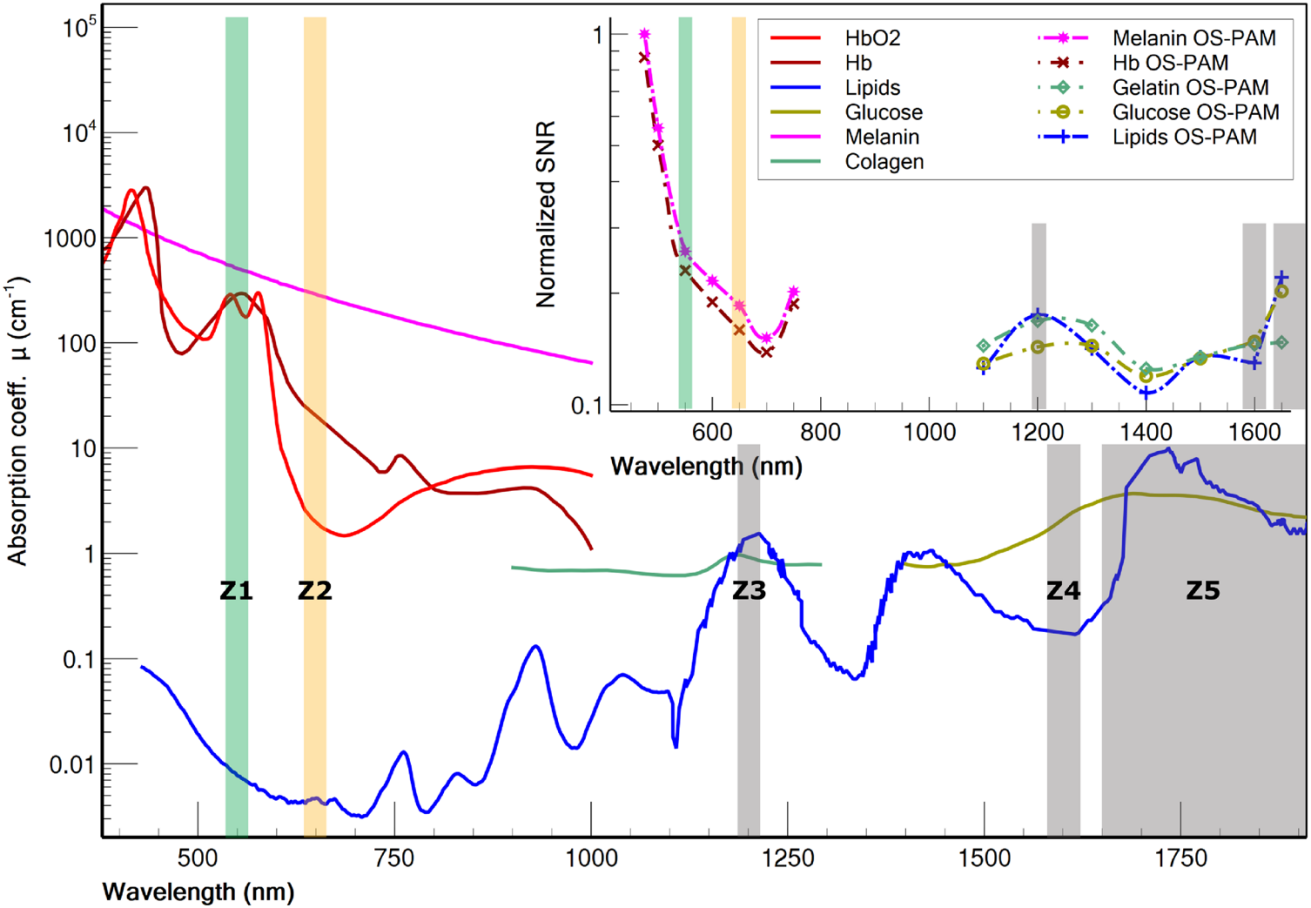
Graphs of the absorption coefficient for the most common endogenous contrast agents in tissue. Data for HBO2, Hb and Melanin were compiled from^31^, data for Collagen from^32^, data for Glucose from^24^, whereas data for lipids from^33^. The spectral windows used for OS-PAM imaging are highlighted in colours. Z1–Z5 represent the spectral zones used for mapping the contrast agents. Inset data is obtained performing in vitro OS-PAM imaging.

The absorption coefficient of the most common endogenous contrast agents in biological tissue as a function of wavelength is presented in Fig. 1. Water is also present in the biological tissue; however, it was not taken into consideration in our procedure as its absorption coefficient, especially in the VIS range, is extremely low, therefore will produce a negligible PAM signal. The spectrum of hemoglobin is presented in Fig. 1 only over the range 450–1000 nm where both HbO2 and Hb are strongly absorbing light and therefore produce a significant PAM signal. The strength of the PAM signal when the sample is illuminated by an optical source operating at a wavelength $\lambda _{i}$ is proportional to the photoacoustic initial pressure amplitude, which can quantitatively be determined using^29^:

1 $$\begin{aligned} p(\lambda _{i})=\Gamma \times \mu _{j} \times \Phi (\lambda _{i}) \end{aligned}$$

Here, $\Gamma$ represents the Grüneisen coefficient, $\mu _{j}$ the optical absorption coefficient of a specific contrast agent, and $\Phi$ is the laser’s irradiance. As, $\Gamma$ exhibits only low variations within the biological media^30^, it is reasonable to consider it as a constant. If a total number of *N* contrast agents are present in the sample, the optical energy is absorbed by all of them, and Eq. (1) can be re-written as,

2 $$\begin{aligned} p(\lambda _{i})=\Gamma \times \sum _{j=1}^{N} \mu _{j} \times \Phi (\lambda _{i}) \end{aligned}$$

As it can be observed (Fig. 1), for any wavelength across the spectral range, typically only 2–3 chromophores contribute significantly towards the photoacoustic signal: melanin and hemoglobin in the visible, water, collagen, lipids in the 1200 nm spectral region, glucose, lipids and water in the 1550–2000 nm region.

For simplicity, let us consider that the sample is illuminated with a wavelength $\lambda _{1}$ then by one of wavelength $\lambda _{2}$ and that only two chromophores *a* and *b* of absorption coefficients, $\mu _{a}$ and $\mu _{b}$, respectively contribute towards the final photoacoustic signal. If we calculate the difference between the initial pressures created by the two chromophores at $\lambda _{1}$ and $\lambda _{2}$ ($\delta p$), by using Eq. (2), the initial pressures created by the radiation at each wavelength can be calculated as,

3 $$\begin{aligned} \left\{ \begin{array}{rcl} p(\lambda _{1}, a) &{} = &{} \frac{\delta p}{\zeta } \\ p(\lambda _{1}, b) &{} = &{} m_{1}\frac{\delta p}{\zeta }\\ p(\lambda _{2}, a) &{} = &{} \alpha _{a}\frac{\delta p}{\zeta }\\ p(\lambda _{2}, b) &{} = &{} m_{2}\alpha _{a}\frac{\delta p}{\zeta } \end{array} \right. \end{aligned}$$

In Eq. (3),

$$\begin{aligned} \left\{ \begin{array}{l} m_{1} = \frac{\mu _{b}(\lambda _{1})}{\mu _{a}(\lambda _{1})},\quad m_{2} = \frac{\mu _{b}(\lambda _{2})}{\mu _{a}(\lambda _{2})}\\ \\ \alpha _{a} = \frac{\mu _{a}(\lambda _{2})}{\mu _{a}(\lambda _{1})}\frac{\Phi (\lambda _{2})}{\Phi (\lambda _{1})},\quad \alpha _{b} = \frac{\mu _{b}(\lambda _{2})}{\mu _{b}(\lambda _{1})}\frac{\Phi (\lambda _{2})}{\Phi (\lambda _{1})} \\ \\ \zeta = 1 + m_{1} - \alpha _{a} - \alpha _{a}m_{2} \end{array} \right. \end{aligned}$$

The wavelengths $\lambda _{1}$ and $\lambda _{2}$ can be selected in such a way that the initial pressure due to chromophore *a* is higher at $\lambda _{1}$, than at $\lambda _{2}$ and the initial pressure due to chromophore *b* is lower at $\lambda _{1}$ than at $\lambda _{2}$. Supposing that only one chromophore contributes to the brightness of a pixel in the image, we compute the difference between images generated at different wavelengths to figure out which of the chromophores is present at each location in the image. If for a given pixel, the difference is positive, the chromophore contributing to the signal is *a*. On the contrary, if the difference is negative, the contributing chromophore is b. Chromophore *a* can for example be Hb and chromophore *b* melanin, $\lambda _{1}=550$ nm (zone Z1 in Fig. 1) and $\lambda _{2} =650$ nm (zone Z2 in Fig. 1). Using the absorption coefficients of the two chromophores at their respective wavelengths, and the values of the optical powers on the sample experimentally measured (0.36 mW at 550 nm and 1.1 mW at 650 nm), we have $m_1=1.83$, $m_2=14.14$, $\alpha _a=0.22$ and $\alpha _b=1.72$. If we take for example $\frac{\delta p}{\zeta } = 1$ arbitrary unit, we obtain,

$$\begin{aligned} \left\{ \begin{array}{rcl} p(\lambda _{1}, a) &{} = &{} 1 \\ p(\lambda _{1}, b) &{} = &{} 1.83 \\ p(\lambda _{2}, a) &{} = &{} 0.22 \\ p(\lambda _{2}, b) &{} = &{} 3.17 \end{array} \right. \end{aligned}$$

So, if in a point of the PAM image we have contributions from both, melanin, and Hb, when switching from 550 to 650 nm we do expect an increase of the initial pressure due to the melanin and a decrease of the initial pressure due to the Hb. Now, if we suppose that from a single point, we have either signal from Hb or melanin then, $\delta p > 0$ indicates the presence of the Hb whereas $\delta p < 0$ that of the melanin.

By carefully selecting the operation wavelength of the instrument, various chromophores can be mapped in the en face (transverse) PAM image. To map Hb and melanin, we used the spectral windows Z1 and Z2, around 550 and 650 nm, respectively. To map glucose, collagen and lipids, three zones were selected (Z3 around 1200 nm, Z4 around 1600 and Z5 above 1700 nm, respectively). Because water is a major absorber at long wavelengths, zones Z3-Z5 were selected in such a way that, when performing the difference between images, one of the targeted chromophores increase (or decrease) its initial pressure from one zone to the other, whereas water and the other chromophore decrease (or increase) their initial pressure. An extensive description of the technique presented in this section is provided in the Supplementary Material.

## Results

### System characterization and OS-PAM/OCT imaging

**Figure 2:**
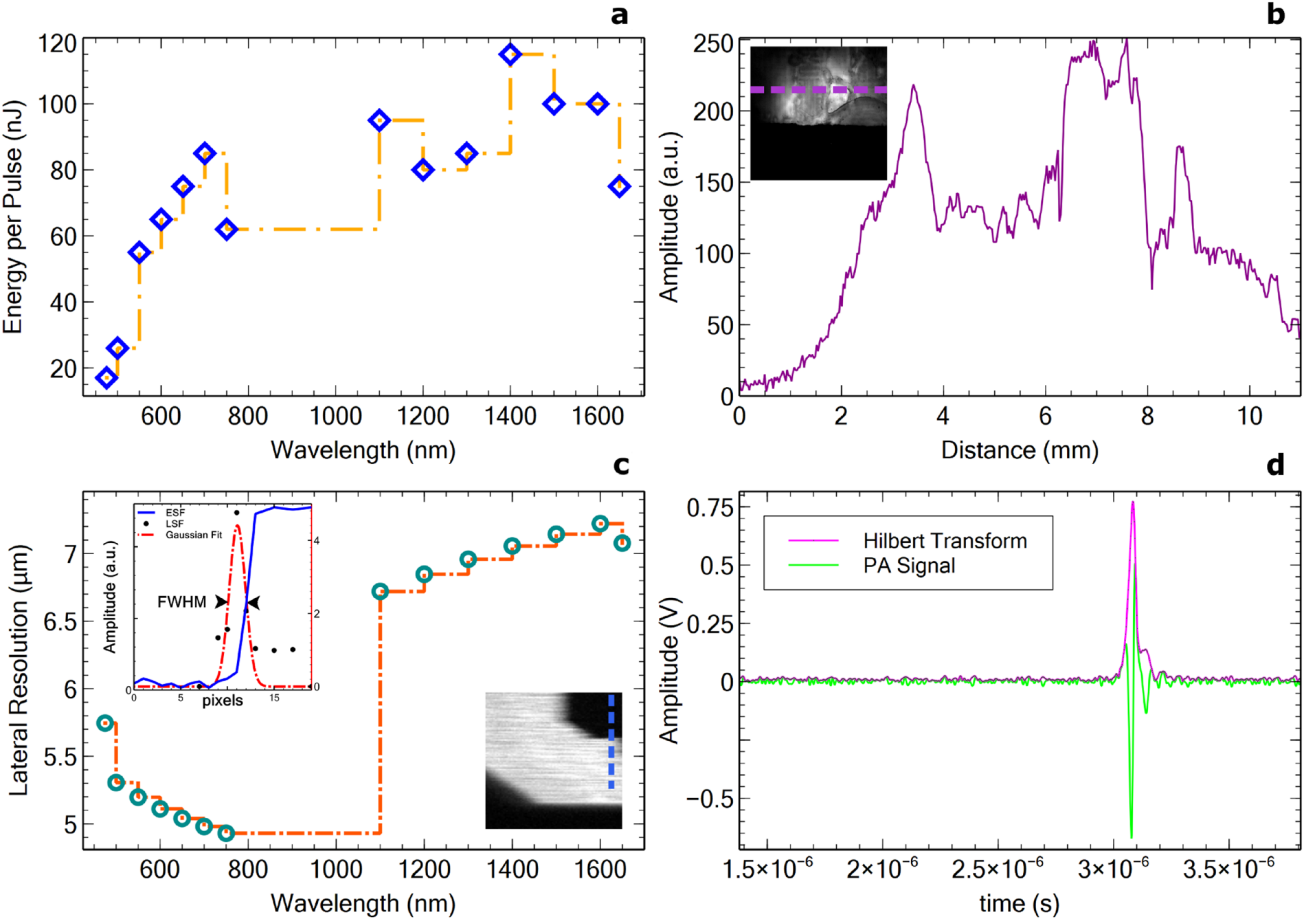
OS-PAM System characterization; (**a**) EPP graph over the whole imaging spectral range measured on the sample; (**b**) Characterisation of the lateral field of view (FOV) of the instrument. PAM signal collected whilst imaging a carbon fibre tape is plotted for each lateral position in the image. The plot corresponds to the amplitudes of the signal at the positions showed by the purple dashed line in the inset image. The size of the image $10\times 10$ mm; (**c**) Lateral resolution per wavelength measured by imaging a sharp edge (letter) on a USAF target, extracting the ESF and calculating the LSF. The size of the image is $0.5\times 0.5$ mm; (**d**) Axial resolution measured imaging carbon fibre tubes. The Axial resolution is defined by the FWHM of the Hilbert transform (Signal envelope).

The OS-PAM system was rigorously characterized and the results are presented in Fig. 2. The EPP was measured on the sample for each wavelength and was found to range from 20 to 110 nJ (Fig. 2a). The FOV was estimated imaging a carbon fibre tape demonstrating high photoacoustic signal amplitude over 8 mm (Fig. 2b). As the lateral resolution varies with the wavelength and the beam diameter it is necessary to be measured for each wavelength. Thus, for each wavelength, a sharp edge of a USAF target (a letter on the USAF target) was imaged. Images of $500\times 400$ $pixels^{2}$ were produced, of size $500\times 400$ μm^2^ therefore, each pixel in the image spans over 1 μm. The edge spread function (ESF) and the line spread function (LSF) were measured and the FWHM of the Gaussian fit to the LSF determined the lateral resolution. The measured lateral resolution, presented in Fig. 2c, was found to vary from 4.9 to 7.1 μm over a spectral range spanning from 475 to 1650 nm. This range of variation in the lateral resolution is expected due to the variation of the beam diameter at the output of the optical source as a function of wavelength (as detailed in the “Methods” part of the manuscript), the beam diameter at the output of the optical source is a function of wavelength. Therefore, if we define the lateral resolution of the instrument as given by 1/2 of the size of the Airy disk^34,35^ and consider the 2$\times$ magnification of the beam expander BE, theoretical lateral resolutions of 5.8 μm at 500 nm (beam diameter 2 mm) and of 6.95 μm at 1500 nm (beam diameter 5 mm) are obtained. These values match the experimental values and ensure that the achromat lens employed did not introduce optical aberrations. The theoretical axial resolution of the OS-PAM system is 38 μm. However, outside the acoustic focal point, the SNR is weaker, and the axial resolution degrades. Furthermore, the transducer has a maximum detectable bandwidth when it is oriented orthogonal to the direction of propagation of the incident acoustic waves. Any small deviation of the incidence angle from 90 degrees, due to the angle directivity dependence, determines a reduction in the detectable signal bandwidth^36^. Hence, the measured axial resolution using the carbon fibre tubes (Fig. 2d) results as 50 μm. The ratio between the max signal amplitude (Fig. 2) and the standard deviation of the noise determines the SNR of the OS-PAM system, found to be 43.8 dB.

The method presented in section “Theoretical background/distinguishing different contrast agents” was experimentally confirmed by producing PAM images showing the spatial distribution of the melanin, hemoglobin, gelatine, glucose, and lipids present in various samples.

**Figure 3:**
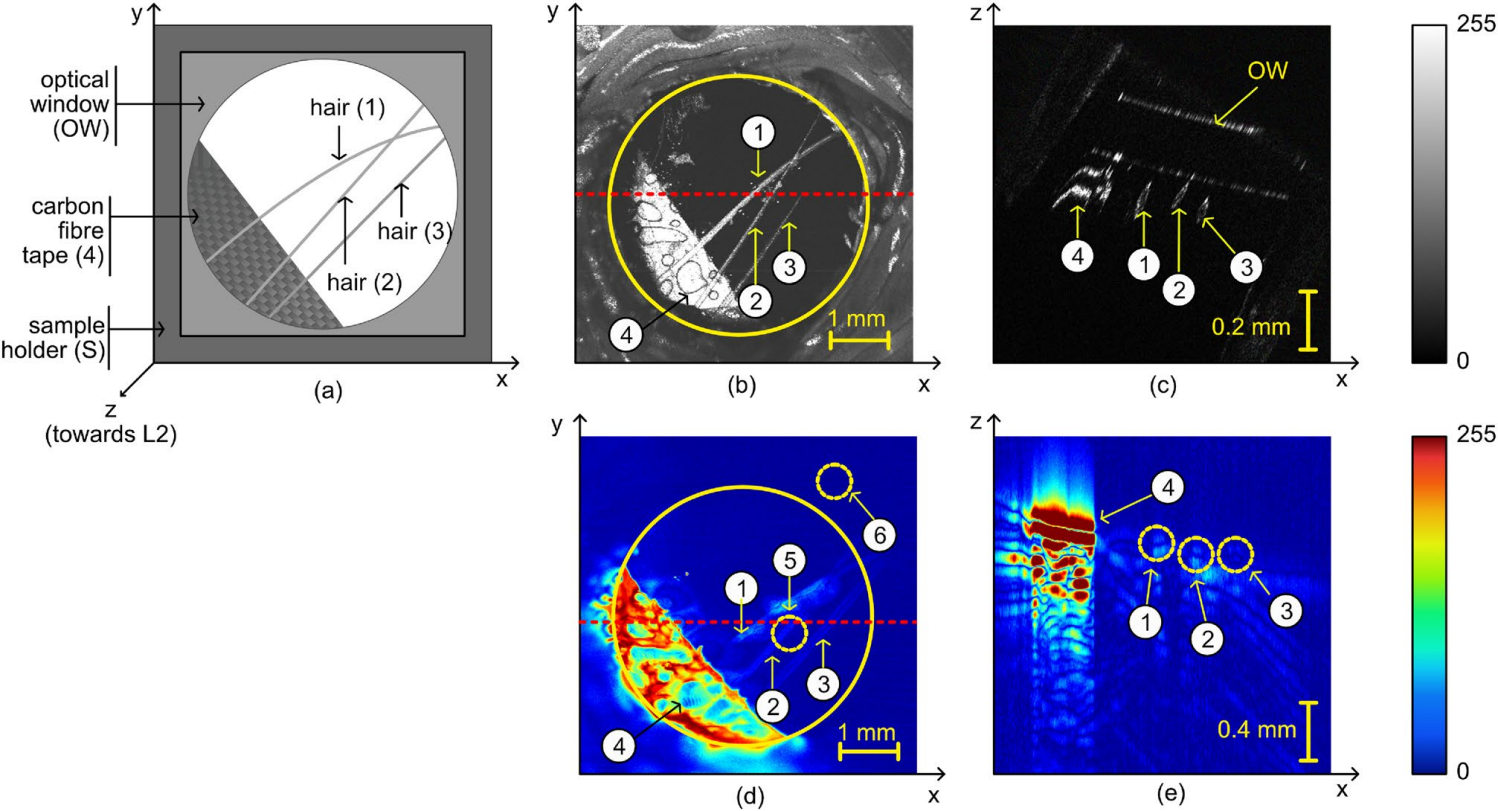
(**a**) Schematic diagram of the sample designed to produce images of the human hair: 1, 2, 3 are the three imaged hairs, placed on an optical window (OW); the ends of the hairs are covered by a carbon fibre tape (4); incident light from OS1 and OS2 travels towards the sample in the *z*-direction. (**b**) En face OCT image of the sample showing both the carbon fibre tape (4) and the three hairs. (**c**) Example of a B-scan OCT image showing the carbon fibre tape, the hairs as well as the optical window. The hairs look elongated as they are not placed orthogonal to the *xz*-plane. (**d**) En face PAM image showing the carbon fibre tape and the hairs. Regions 5 and 6 are used to calculate the signal-to-noise ratio using the procedure described within the manuscript. (**e**) Example of a B-scan PAM image. The optical window is not visible in the PAM image. The red horizontal dashed lines shown on the en face images indicate the y-position where the B-scans are originating from.

To map the spatial distribution of melanin, three human hairs were placed in the 3D sample holder S (please see Fig. 7), on the optical window, and illuminated from below using the instrument presented in section “OS-PAM/OCT system”. To guide the imaging operation, and hold the hairs in place, a carbon fibre tape partially covered the hairs, as shown in the 2D schematic diagram of the sample presented in Fig. 3a. The three hairs are labelled as 1, 2 and 3, whereas the carbon fibre tape as 4.

Optical source OS1 is used to sequentially illuminate the sample with light at 550 nm and then at 650 nm to generate PAM maps of the chromophores producing acoustic waves when excited by light at these two wavelengths. Subsequently, by using the procedure introduced in section “Theoretical background/distinguishing different contrast agents”, PAM spatial distribution maps of the melanin were produced. In addition, after producing the maps of melanin, the optical source OS2, operating at a central wavelength of 1300 nm, was used to illuminate the sample to generate OCT images.

OCT and PAM images hence obtained are presented in Fig. 3b–e. In Fig. 3b,d, en face OCT and PAM images are shown, whereas in Fig. 3c,e, examples of B-scan OCT and PAM images respectively. The images are presented in 8-bit format, therefore, as illustrated in their corresponding colormap bars, the brightness 0 of a pixel in the image corresponds to an OCT or PAM signal equal to zero, whereas a brightness of 255 to its maximum value. The en face images were produced by using the maximum intensity projection algorithm. As expected, the carbon fibre tape is visible in both OCT and PAM images (areas 4) and very important, all three hairs are clearly identifiable in both images, which proves that the procedure suggested here to map the melanin is effective. The B-scan views show, as expected, that the axial resolution in the OCT image is better than in the PAM one.

**Figure 4:**
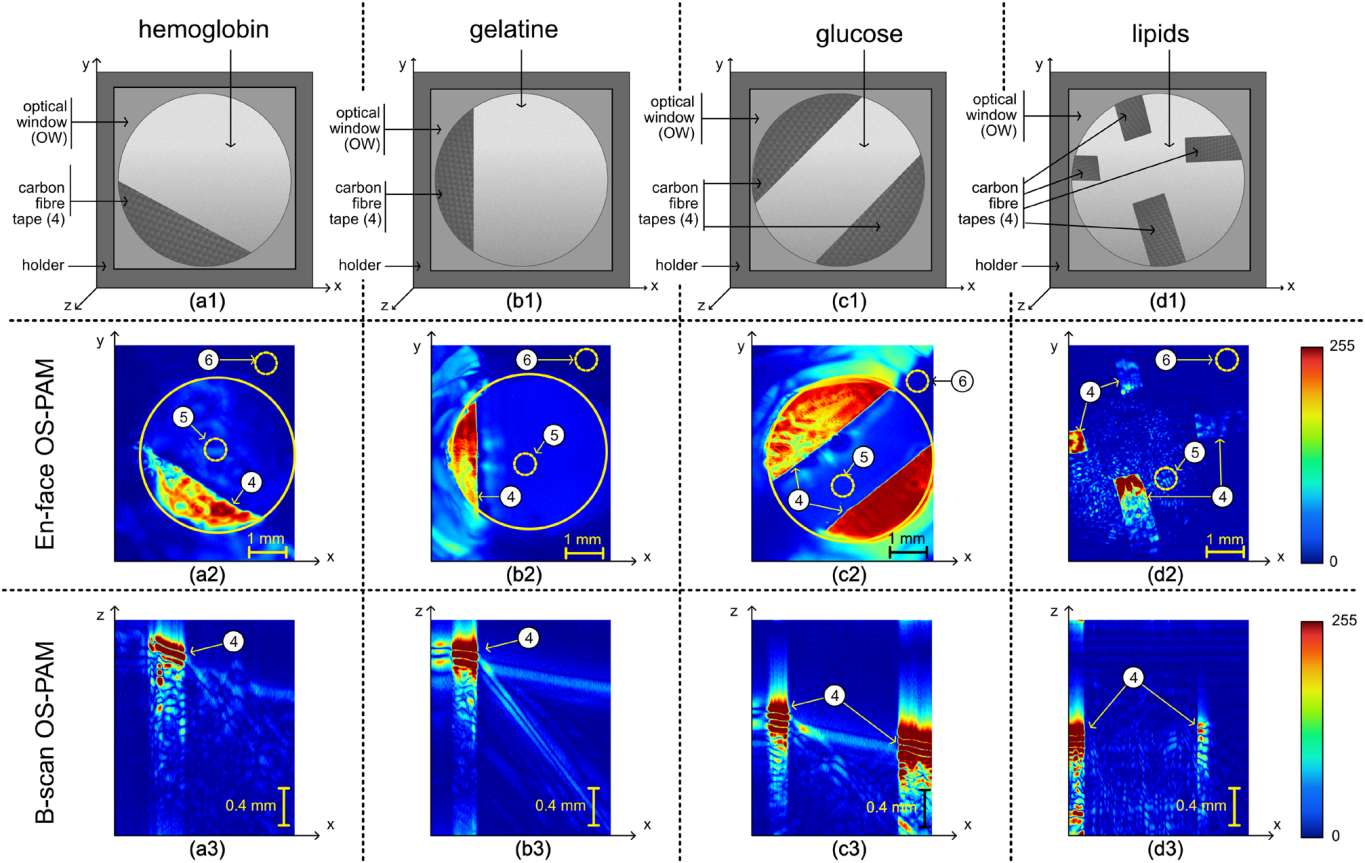
(**a1**), (**b1**), (**c1**) and (**d1**) Schematic diagrams of the samples designed to produce PAM spatial distribution maps of: hemoglobin, gelatine, glucose, and lipids respectively. In all cases, carbon fibre tapes (4) are placed on the optical window (OW) and covered by the liquid mixture created. (**a2**), (**b2**), (**c2**) and (**d2**) En face PAM maps showing the presence of hemoglobin, gelatine, glucose, and lipids respectively. Regions 5 and 6 are used to calculate the signal-to-noise ratio using the procedure described within the manuscript. (**a3**), (**b3**), (**c3**) and (**d3**) Examples of B-scan PAM images of hemoglobin, gelatine, glucose, and lipids respectively. All images show the carbon fibre tapes (4).

To validate the potential of the method proposed to be used to generate spatial distribution maps of hemoglobin, gelatine, glucose, and lipids, mixtures of these chromophores with water were created. The mixtures were placed in the sample holders S, and illuminated with light of various wavelengths, from below, as explained in section “OS-PAM/OCT system”. For imaging guidance purposes, carbon fibre tapes have been placed in the holder, on the optical window (the liquid mixtures would cover the carbon tape). In Fig. 4a1,b1,c1,d1, 2D schematic diagrams of the four samples so created are presented showing the position of the carbon fibre tapes in each of the four cases.

(i) To validate the potential of the technique to map the spatial distribution of hemoglobin, a mixture of dry hemoglobin and water (concentration 150 g/l) was placed in the sample holder, and sequentially illuminated with light from OS1, first at 550 nm and then at 650 nm. The two PAM images so created were subsequently used to generate distribution maps of hemoglobin. An en face PAM distribution map of hemoglobin is presented in Fig. 4a2 whereas an example of a B-scan map in Fig. 4a3.
(ii) To map the spatial distribution of gelatine, a mixture of cooking gelatine and water (concentration 150 g/l) was created, placed in the sample holder, and sequentially illuminated with light from OS1 at 1200 nm and then at 1700 nm. The two PAM images so created were subsequently used to generate gelatine distribution maps. An en face PAM distribution map of the gelatine is presented in Fig. 4b2 whereas an example of a B-scan in Fig. 4b3.
(iii) To produce glucose spatial distribution maps, a mixture of glucose and water (concentration 150 g/l) was created, placed in the sample holder, and sequentially illuminated with light from OS1 operating at 1600 nm and then at 1700 nm. The two PAM images so created were subsequently used to generate PAM glucose distribution maps. An en face PAM map of the glucose is presented in Fig. 4c2 whereas an example of a B-scan map in Fig. 4c3.
(iv) To generate PAM spatial distribution maps of the lipids, a mixture of water and chicken adipose fat has been created, placed in the sample holder, and sequentially illuminated with light from OS1 at 1600 nm and then at 1700 nm. The two PAM images hence created were subsequently used to generate PAM lipids distribution maps. A PAM en face distribution map of the lipids is presented in Fig. 4d2 whereas an example of a B-scan map in Fig. 4d3.

In all cases, en face PAM images are created via a maximum projection algorithm. A summary of the content of the samples created and the wavelengths used in each case to produce PAM spatial distribution maps of the 5 chromophores targeted in our experiments is presented in Table 2.

**Table 2:** Samples created to validate the proposed technique and the wavelengths at which they were sequentially illuminated using OS1 to generate PAM images and subsequently map the chromophores.

Chromophore targeted	Content sample	$\lambda _{1}$ (nm)	$\lambda _{2}$ (nm)
Melanin	Human hair	550	650
Hemoglobin	Water and dry hemoglobin (150 g/l)	550	650
Gelatine	Water and gelatine (150 g/l)	1200	1700
Glucose	Water and glucose (150 g/l)	1600	1700
Lipids	Water and chicken adipose fat	1200	1600

For all imaged chromophores, the values of the signal-to-noise ratio (SNR), normalised over the optical fluence, presented in the inset of Fig. 1 are consistent with the values of their absorption coefficients reported in the literature. To compute the SNR, we used a standard procedure^13,17^ consisting in calculating the SNR as,

4 $$\begin{aligned} SNR=20\log _{10}\left[ \frac{\text {MAX(area 5)}}{\text {STD(area 6)}}\right] \end{aligned}$$

In Eq. (4), MAX(area 5) and STD(area 6) are the maximum value of the PAM signal computed in area 5 and the standard deviation of the signals calculated over region 6 respectively. Regions 5 and 6 are all depicted using dotted yellow circles on the en face PAM images shown in Figs. 3 and 4 .

### In vivo whole SC range OS-PAM imaging of Tadpoles

**Figure 5:**
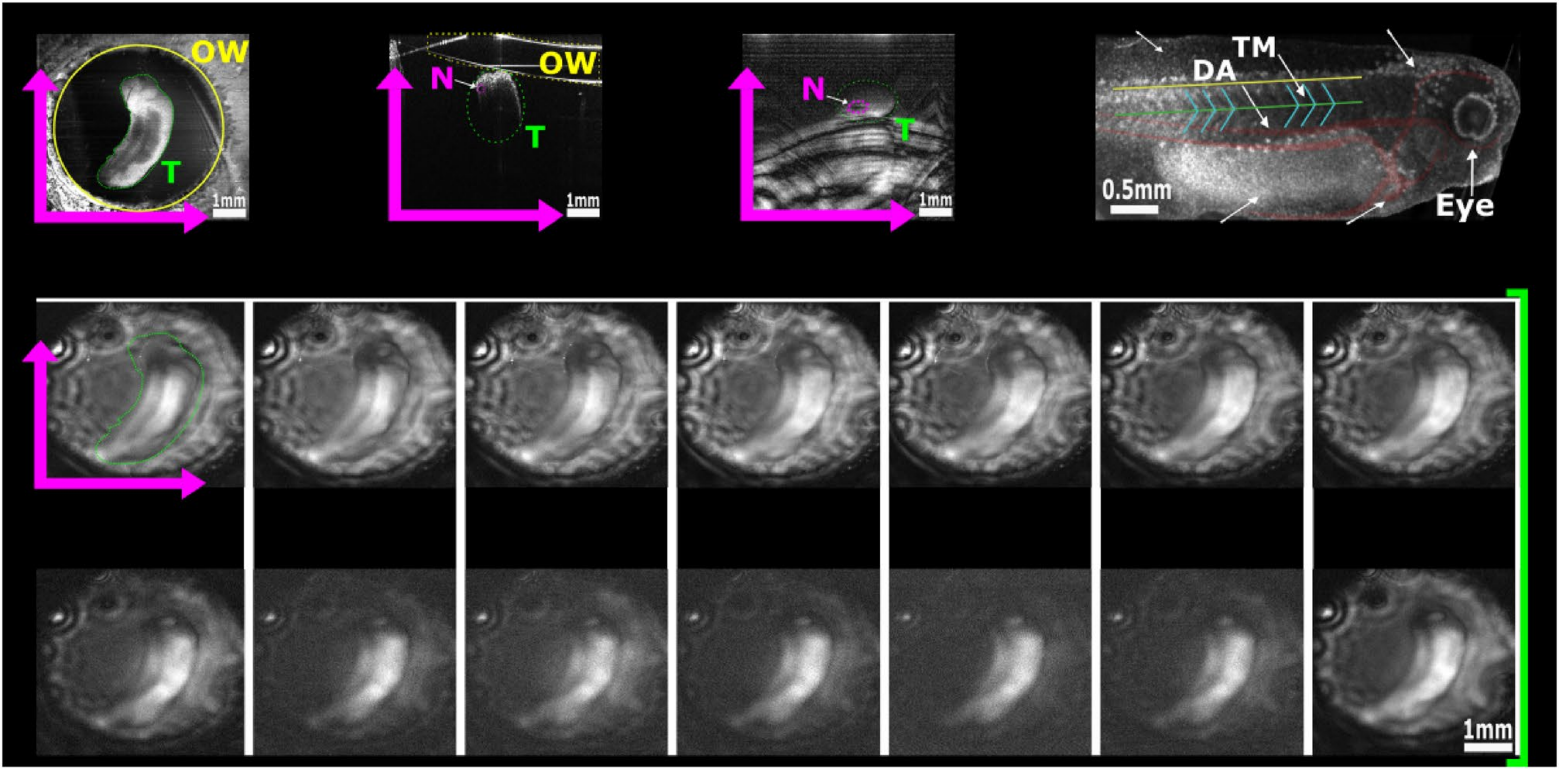
Representative in vivo OS-PAM en face images of one tadpole (T) generated at various wavelengths across the whole emission spectrum of the OS1. High noise levels can be observed above 1200 nm as water absorption increases. The OCT image has a wider field of view providing an overview of the optical window (OW) and the positioning of the tadpole; Top right: Structural en face OCT image displaying tadpole anatomy with highlighted main veins and aortas (red) as well as, the trunk muscles (blue). The green line indicates the position of the notochord (N) while the yellow line the position of the spinal cord. *DF* dorsal fin, *YS* yolk sack, *V* ventricle. This procedure was repeated on four animals (n = 4).

During the OS-PAM imaging, animals were anaesthetized (n = 4, as described in the “Methods” section) and positioned on a 3D printed sample holder. The sample holder was designed to keep the animal submerged in MS-222 solution, while the laser beam scanned the animal through a thin (0.22 mm) glass optical window from below (inset in Fig. 7). For OS-PAM imaging at different spectral bands, 12 hard coated band-pass filters, each of them of 25 nm bandwidth, were employed, enabling imaging from 475 nm up to 1600 nm. At 1600 nm the only commercially available filter to us had 50 nm bandwidth, whereas a long-pass filter with a cut off at 1650 nm was employed for imaging at longer wavelengths. In both OCT and PAM channels, B-scan images of $500 \times 400$ pixels were acquired and displayed in real-time at a frame rate of 20 Hz, hence a 500$\times$400$\times$400 3D volume was generated in 20 s (10 s to capture data and 10 s to process). To enhance the Signal to Noise Ratio (SNR) in the PAM images, 32 A-scans were averaged, increasing the acquisition time to 10.7 min. In Fig. 5, en face z-projected OS-PAM images over the whole SC range are presented, along with a structural en face OCT image clearly showing tadpole’s anatomy.

**Figure 6:**
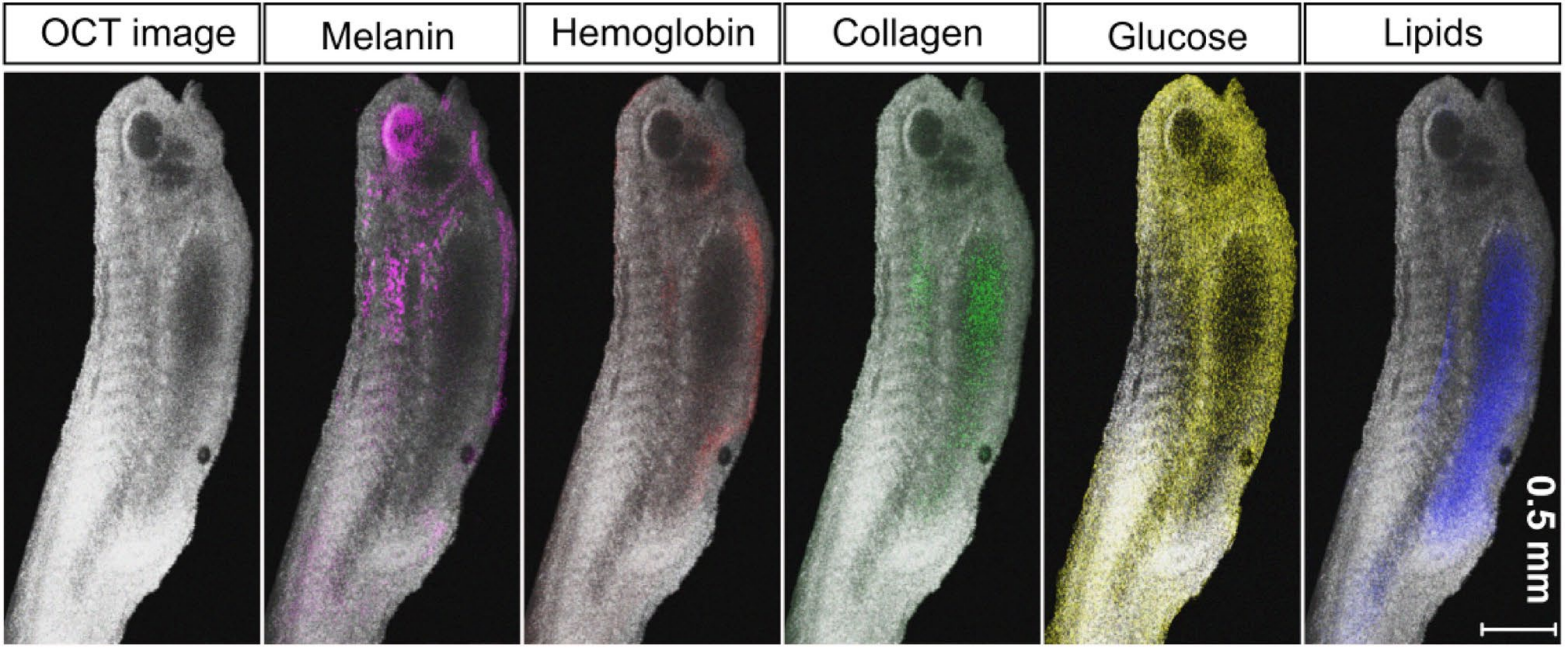
Qualitative illustrations of the superposition of the spatial mapping distribution of melanin (in pink), hemoglobin (magenta), collagen (green), glucose (yellow) and lipids (blue) within a tadpole obtained using the technique proposed, over a structural OCT image of the same tadpole. Similar in vivo images were obtained when imaging other four tadpoles. The image on the left shows the bare OCT image of the tadpole over which the maps of the chromophores were overlaid.

Due to the unavailability of commercial band-pass filters of 25 nm bandwidth at wavelengths over 1650 nm, a long-pass filter with a cut-off at 1650 nm was employed, hence the notation $>1650$ nm. This means that the spectral range used here is from 1650 to 2400 nm, allowing for absorption of chromophores with absorption over a much wider spectral range. Thus, brighter images at for the Z5 zone were obtained. The lateral resolution of 5–7 μm refers to the capabilities of the system when the distance between two consecutive points (pixels) in the image is smaller than the lateral resolution of the system. However, in Figs. 3, 4, 5, 6 we show very large size areas (5–8 mm) while keeping a quite low number of lateral points (500 pixels). Therefore, the lateral resolution was digitally degraded in these images to 10–16 μm respectively.

### Mapping of five endogenous contrast agents with OS-PAM/OCT

Utilising the capabilities of the OS-PAM/OCT instrument in combination with the technique described in section “Theoretical background/distinguishing different contrast agents”, five endogenous contrast agents: melanin, Hb, collagen, glucose and lipids were mapped on four tadpoles at developmental stage 37/38^37^.

The tadpole OS-PAM images were overlaid on the corresponding structural OCT images and presented in Fig. 6. Maps of melanin are produced using filters operating at 550 and 650 nm. On the tadpole, at developmental stage 37/38, pigmentation (due to pigment cells) is present in the eye, on the head and on the dorsal side of the yolk sac (Fig. 5). Moreover, melanophores appear on the tail which is shown by the less intense melanin signal in Fig. 5. Maps of Hb are produced using filters operating at 550 and 650 nm. Hb appears at the level of the cardiac ventricle, as well as, along the developing vascular system which spans the yolk sac and travels along the side of the tail (Figs. 5 and 6). Maps of collagen are obtained by changing to filters operating 1200 and 1700 nm. Collagen has been detected on several areas of the tadpole’s body and can be detected on the developing cranial structures and at the levels of the yolk sac and trunk muscles (Fig. 6). To map glucose, images were produced at 1600 and 1700 nm. High levels of glucose are present within the yolk sac area (Fig. 6) overlapping with the high concentration of lipoproteins in this region^38^. Finally, we used filters at 1200 and 1600 nm to map lipids. Our results show that lipids are highly concentrated in the yolk sac (Fig. 6), which is consistent with previous studies performing multi-spectral PAM imaging on zebrafish and tadpoles^13,17^. A more detailed visual illustration on how the technique was implemented to obtain the overlayed images is presented in Fig. S1 (Supplementary Material).

## Discussion and conclusion

A PAM imaging instrument operating at wavelengths over the entire spectral range of a commercial SC source, from 475 to 2400 nm, is demonstrated. This is employed on mapping five endogenous contrast agents in living tadpoles, namely melanin, hemoglobin, collagen, glucose, and lipids. Based on the supposition that only one chromophore contributes to the photo-acoustic signal of each individual voxel in the 3D PAM image, a novel technique of mapping absorbers is demonstrated. A sequentially operating ultrahigh resolution OCT imaging channel aids the investigation.

To compensate for the limited optical power delivered by the SC source employed here, losses and optical aberrations were minimized by a careful selection of the optical components used to convey light from the source, to the sample. As a result, the experimentally measured EPP on the sample ranged from 17 to 110 nJ, over the whole spectral range, hence sufficiently strong photo-acoustic signal could be generated. The main advantage of the PAM imaging method demonstrated here is its versatility in performing spectral measurements in the range of interest without resorting to multiple optical sources or changes in its optical design.

Our OS-PAM instrument, in its current stage of development, is not capable of performing spectroscopic PAM, therefore the results obtained are not directly comparable to conventional spectral unmixing techniques. By using the suggested procedure, the concentration of the targeted chromophores cannot be extracted quantitatively but only their spatial mapping distribution evaluated. Adequate experimental assessments require quantitative concentration measurements. Therefore, this was not the goal of the research presented here. Instead, the focus was on using a unique approach for the spatial distribution of the chromophore. In doing so, two milestones were targeted: (i) PAM imaging over an unprecedented spectral range using an ultra-broadband source and (ii) utilizing PAM imaging to map chromophores of bio-medical interest in vivo, in several spectral regions. These two milestones were targeted despite facing the issues of a low energy per pulse and intrinsic high noise challenges of a supercontinuum source. However, such sources are commercially available and utilisation of a single source for OCT and PAM as illustrated may represent a cost-effective solution. Although the technique proposed here cannot be used currently to produce quantitative absorption coefficient data, it shows great promise in detecting the presence of a plethora of chromophores, without limitations due to the restricted operational spectral range of the conventional sources employed in PAM.

The technique presented here has been successfully employed to detect the presence of chromophores using a particular Supercontinuum optical source and a particular design of the optical setup. Therefore, what it is important when detecting a specific chromophore, is not the absorption coefficient of a certain chromophore at a certain wavelength alone but also the fluence and the spectral density variation of the source. Therefore, the two interrogation wavelengths must be selected very carefully. The spectral region from 700 to 1100 nm, although of interest for biomedical applications, is not targetable by PAM due to the very low absorption of the chromophores, for which reason we did not perform any measurements over this range. By avoiding this, we also kept the project cost effective. As the diameter of the optical beam is a function of wavelength (intrinsic characteristic of the optical source), the lateral resolution of the system does not increase linearly with the wavelength. Consequently, although, the lateral resolution should deteriorate as we move to longer wavelengths, the size of the beam diameter expands, increasing the numerical aperture, and therefore improving the lateral resolution (Fig. 2c).

Although more sophisticated systems are capable of obtaining accurate absorption coefficient data, they are either limited to two chromophores due to lack of spectral coverage, or they cannot perform in vivo imaging due to lack of imaging speed (Table 1). We acknowledge that the rate at which images are produced in this study is comparable to that of the OPOs instrument. However, as our implementation is based on fast acquisition of cross-sectional slices, it is better suited for in vivo imaging.

A low-cost PAM imaging instrument could be devised with potential in various medical screening programs. Future work includes increasing the acquisition sensitivity of the OS-PAM instrument for faster acquisition by replacing the ultrasound transducer (UT) with a higher sensitivity focused transducer, applying lower noise amplification methods, implementing software noise reduction techniques, and replacing the band-pass filter wheel with and automated one, synced with the acquisition. For this proof of concept study, a transmission mode PAM was considered in order to minimize imaging artefacts and provide maximum achievable SNR. As the next step of these studies is to image BCC and melanoma in vitro, with the ultimate goal to monitor these notorious skin lesions in patients (clinical applications), the probe would require to be redesigned for reflection mode. We are confident that our system has the potential to tackle several challenges of skin cancer diagnosis. In melanoma for instance, the progression of melanocytes to subsequent pathological growth phases^1^ can be monitored by mapping melanin in the epidermis. D’Alessandro et al.^2^ demonstrated that multi-spectral imaging can be used to quantify saturated Hemoglobin ($HbO_2$), which is a major indicator for the early detection of melanomas whereas Fang et al.^3^ showed how collagen can inhibit and promote tumor progression. Long et al.^4^ showed that energy metabolism, especially lipid metabolism, is significantly elevated during carcinogenesis. Furthermore, various studies also demonstrated that cell metabolism is highly dysregulated in cancer, as lipids and glucose become sources for tumor progression via multiple signaling pathways^5–8^. Although the localization in real-time of multiple endogenous chromophores in tissue seems to be of paramount importance, the available technology does not allow simultaneous detection of all the physiological changes listed above.

## Methods

### Ethical approval

Animals and Ethical Approval In vivo imaging was performed on four *Xenopus laevis* tadpoles at developmental stage 37/38, based on Nieuwkoop and Faber 1956. All experimental procedures were approved by the University of Kent Animal Welfare and Ethical Review Body (AWERB; Institutional Ethics Reference Number: 0037-SK-17). Reporting of all in vivo experimental work conforms with the ARRIVE guidelines.

*Xenopus laevis* embryos were supplied by the European Xenopus Resource Center (EXRC; Portsmouth UK). The Xenopus embryos were kept in water at 20 C until they reached the developmental stage 37/38^37^. Prior to OS-PAM imaging, animals were anesthetized in $0.1\%$ MS-222 solution (ethyl 3-aminobenzoate methane sulfonate, Sigma-Aldrich UK^39^. Animals remained anesthetised in MS-222 solution for the entire duration of the imaging procedure (4.2 min). All methods employed were performed in accordance with guidelines and regulations as described in the research protocol approved by the University of Kent Animal Welfare and Ethical Review Body.

### OS-PAM/OCT system

The schematic diagram of the multi-modal OS-PAM/OCT imaging instrument is presented in Fig. 6. In the OS-PAM channel, a SC source (SuperK COMPACT, NKT Photonics) delivering pulses of 2 ns duration at 20 kHz PRR (maximum) is employed (OS1). To improve imaging lateral resolution, the size of the beam diameter was doubled by using a reflective beam expander BE (BEO2R/M, Thorlabs), as the spot size is proportional to the focal length and inversely proportional to the beam diameter. Sequentially, a smaller spot size means higher fluence (same energy over a smaller area), resulting in an improvement of SNR.

The lateral resolution per wavelength is presented in Fig. 2c. This is important especially at shorter wavelengths, as the output beam diameter delivered by the COMPACT source varies from 1 mm in the visible up to 3 mm in the near-infrared region. As the numerical aperture is determined by the diameter of the beam, whose size is determined by the wavelength employed, measurements of the lateral resolution of the OS-PAM system are presented for each operational wavelength (Fig. 2c). Doubling the beam diameter, means smaller spot size, thus the optical energy is delivered in a smaller area which is crucial for obtaining good quality PAM images when using a laser with low EPP (Fig. 2) such as the COMPACT.

To optimize the optical power on the sample, optical components were carefully selected in such a way that the beam wave-front is not disturbed by chromatic aberrations. To this aim, the only optical components in the beam’s path are a reflective beam expander, a flipping silver coated mirror (FM, used to switch between the PAM and the OCT imaging modes), the 25 nm bandwidth hard coated band-pass filters (87-776, Edmund Optics), a pair of orthogonal galvo-scanners (6220H, Cambridge Technology) and a 19 mm focal length achromatic doublet (AC127-019-AB) as objective lens. When using achromatic doublets, due to the fact that the anti-reflection coating is effective only over a limited spectral range, losses can potentially be significant. However, the EPP presented in Fig. 2a was measured on the sample which includes the losses coming from the lens. In addition, the experimental lateral resolution measured did not change dramatically along the spectral range, therefore no, or very little aberrations were observed, which is evident from the images presented in Fig. 4. All these components attenuate the optical power by 13%. Switching between filters takes about 2–5 s, however this procedure can be faster by using PC controlled filters.

**Figure 7:**
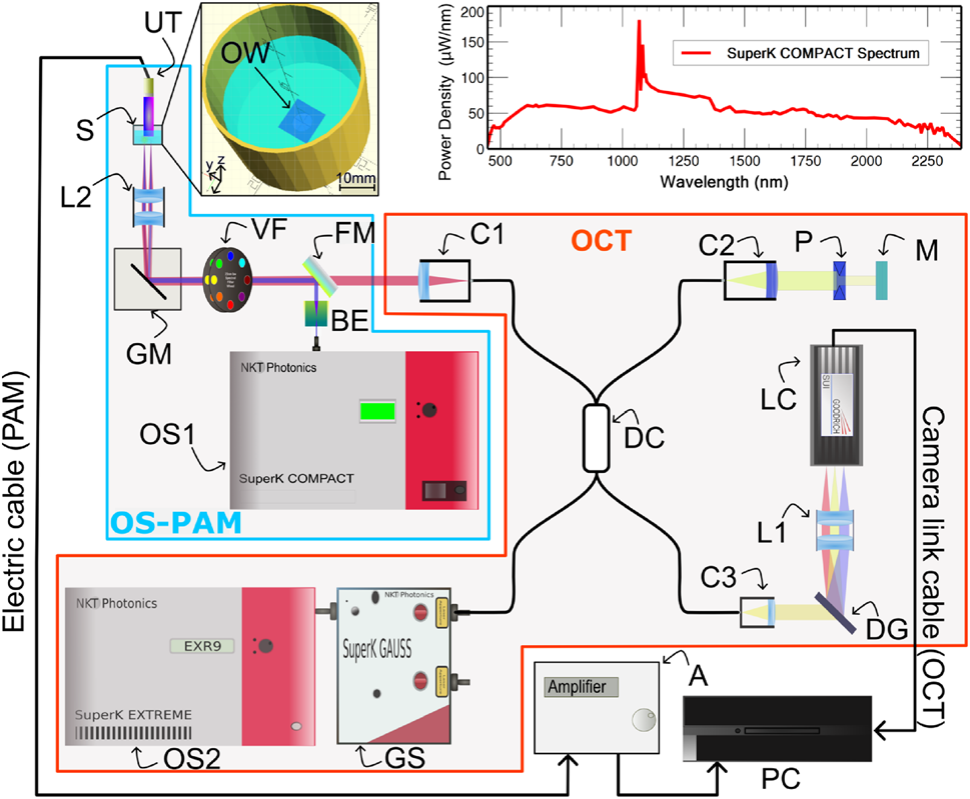
Schematic diagram of the OS-PAM/OCT system. *OS1* OS-PAM excitation optical source (SuperK COMPACT, NKT Photonics), *OS2* OCT optical source (SuperK EXTREME EXR9, NKT Photonics), *GS* Variable filter (SuperK GAUSS, NKT Photonics), *VF* Bandpass filter wheel, *BE* Beam expander, *FM* Flipping mirror, *GM* X–Y galvanometer mirrors, *LC* Line camera, *DG* Diffraction grating, *DC* 50/50 directional coupler, *UT* Ultrasound transducer, *S* Sample, *C1–C3* Collimators, *L1–L2* Lenses, *M* Flat mirror, *P* Pinhole, *A* Amplifier, *OW* Optical window of the 3D printed sample-holder; Drawing produced using Incscape 1.0.1 (https://inkscape.org/).

The sample is submerged in water to facilitate acoustic coupling. The acoustic waves are detected with a high frequency customized unfocused needle ultrasonic transducer (40.3 MHz center frequency, 90 % bandwidth at 6 dB, 0.4 mm diameter active element, University of Southern California) which provides an axial resolution of 50 μm (Fig. 2d). This is placed in contact with water. At the bottom of the 3D printed sample (S in Fig. 7) a circular optical window (OW) of 0.22 mm thickness is placed. The sample is sitting on the optical window, being illuminated from below. The OW does not absorb optical energy, therefore is invisible in all OS-PAM images. The electrical signal generated is then amplified by two low noise wide-band amplifiers (ZFL-500LN+, Mini Circuits) and then digitized using a 12-bit fast acquisition board operating at a sampling rate of 200 MS/s (PCI-5124, National Instruments).

The COMPACT (OS1) could potentially be used as a light source for the OCT channel as well, as demonstrated in^23,40^. As the goal of this paper is to demonstrate OS-PAM system’s capabilities, the OCT subsystem was implemented only to guide the OS-PAM imaging and to provide structural information. The need for multi-modality imaging instruments has been emphasised in several studies^41,42^. Thus for the OCT channel, a second low noise supercontinuum laser (SuperK EXTREME EXR9, NKT Photonics) (OS2) was employed. OS2 is coupled into a tunable filter (SuperK Gauss, NKT Photonics), using its IR channel (central wavelength 1310 nm and spectral bandwidth 180 nm). The state-of-the-art ultrahigh resolution OCT instrument is used sequentially with the OS-PAM. Light from OS2 is directed towards the galvanometer scanner head (GM), then conveyed through the objective to the sample. Light back-scattered by the sample returns into the 50/50 directional coupler (DC) being directed towards the spectrometer. The spectrometer consists of a custom made collimator, a transmission diffraction grating (Wasatch Photonics), a doublet pair as a focusing lens and a line camera (LC, SU1014-LDHI, Goodrich,) with 1024 pixels and 25 μm pitch, 47 kHz max reading rate, here operated at 20 kHz. Data is digitized using a camera link board (National Instruments, model IMAQ 1429).

The display of B-scan OCT and PAM images is performed in real-time. The generation of the PAM A-scans does not involve complex mathematical operations (a Hilbert transform is applied to each acquired temporal signal), hence the real-time display of the images is straightforward. The OCT channel is powered by the Master-Slave procedure, which allows for generation of direct en face views and of A-scans, with no need for resampling/linearisation of data^43,44^.

An in-house LabVIEW software was developed to drive the acquisition and the analysis procedure. Thus, the PCI 5124 digitizer was employed to digitize the electrical signal generated by the ultrasound transducer in synchronism with the pulses generated by OS1. A-scans from the signals generated by both PAM and the OCT channels are produced for each position of the scanned beam during the lateral scanning of the sample.

## Supplementary Information

Supplementary Information.

## Data Availability

All data generated or analysed during this study are included in this published article (and its Supplementary Information files).
