# Supplementary material for: Two octaves spanning photoacoustic microscopy
Source: Sci Rep. 2022 Jun 22;12:10590. doi: 10.1038/s41598-022-14869-5 (PMC9218110; doi:10.1038/s41598-022-14869-5)
Supplement: Supplementary file 1 — Supplementary Information. [file 41598_2022_14869_MOESM1_ESM.pdf]

Two Octaves Spanning Photoacoustic Microscopy - Supplementary Material

Gianni Nteroli[a,†,*], Manoj K. Dasa[b,†], Giulia Messa[c], Stella Koutsikou[c], Magalie Bondu[d], Peter M. Moselund[d], Christos Markos[b], Ole Bang[b], Adrian Podoleanu[a], and Adrian Bradu[a]

[a]Applied Optics Group, University of Kent, Canterbury, UK.
[b]DTU Fotonik, Technical University of Denmark, 2800 Kgs. Lyngby, Denmark.
[c]Medway School of Pharmacy, University of Kent, Chatham, UK.
[d]NKT Photonics A/S, Blokken 84, 3460 Birkerød, Denmark.
[*]G.Nteroli@kent.ac.uk
[†]these authors contributed equally to this work

ABSTRACT

Theoretical background / Distinguishing different contrast agents

In this section, a detailed demonstration of the formulae used to calculate the initial pressures produced by two chromophores at two different wavelengths (Eqs. 3 in the main manuscript) is presented. To this goal, let us suppose that the initial total pressures at wavelengths λ_1 and λ_2 created by two chromophores a and b are respectively,

$$\begin{cases} p_0(\lambda_1) = \Gamma[\mu_a(\lambda_1) + \mu_b(\lambda_1)]\Phi(\lambda_1) \\ p_0(\lambda_2) = \Gamma[\mu_a(\lambda_2) + \mu_b(\lambda_2)]\Phi(\lambda_2) \end{cases} \tag{1}$$

By performing the difference between the two equations above:

$$p_0(\lambda_1) - p_0(\lambda_2) = [\Gamma\mu_a(\lambda_1)\Phi(\lambda_1) + \Gamma\mu_b(\lambda_1)\Phi(\lambda_1)] - [\Gamma\mu_a(\lambda_2)\Phi(\lambda_2) + \Gamma\mu_b(\lambda_2)\Phi(\lambda_2)] \tag{2}$$

It follows that:

$$p_0(\lambda_1) - p_0(\lambda_2) = [\Gamma\mu_a(\lambda_1)\Phi(\lambda_1) - \Gamma\mu_a(\lambda_2)\Phi(\lambda_2)] + [\Gamma\mu_b(\lambda_1)\Phi(\lambda_1) - \Gamma\mu_b(\lambda_2)\Phi(\lambda_2)] \tag{3}$$

The equation above can be written as:

$$p_0(\lambda_1) - p_0(\lambda_2) = P_a + P_b \tag{4}$$

where,

$$\begin{cases} P_a = \Gamma\mu_a(\lambda_1)\Phi(\lambda_1) - \Gamma\mu_a(\lambda_2)\Phi(\lambda_2) = \Gamma\mu_a(\lambda_1)\Phi(\lambda_1)\left[1 - \dfrac{\Gamma\mu_a(\lambda_2)\Phi(\lambda_2)}{\Gamma\mu_a(\lambda_1)\Phi(\lambda_1)}\right] = p_0(\lambda_1, a)\left[1 - \dfrac{\mu_a(\lambda_2)\Phi(\lambda_2)}{\mu_a(\lambda_1)\Phi(\lambda_1)}\right] \\ P_b = \Gamma\mu_b(\lambda_1)\Phi(\lambda_1) - \Gamma\mu_b(\lambda_2)\Phi(\lambda_2) = \Gamma\mu_b(\lambda_1)\Phi(\lambda_1)\left[1 - \dfrac{\Gamma\mu_b(\lambda_2)\Phi(\lambda_2)}{\Gamma\mu_b(\lambda_1)\Phi(\lambda_1)}\right] = p_0(\lambda_1, b)\left[1 - \dfrac{\mu_b(\lambda_2)\Phi(\lambda_2)}{\mu_b(\lambda_1)\Phi(\lambda_1)}\right] \end{cases}$$

In the equations above,

$$p_0(\lambda_1, a) = \Gamma\mu_a(\lambda_1)\Phi(\lambda_1) \text{ and } p_0(\lambda_1, b) = \Gamma\mu_b(\lambda_1)\Phi(\lambda_1)$$

are the initial pressures created by the two chromophores at λ_1.

$$\begin{cases} P_a = p_0(\lambda_1, a) \cdot \left[1 - \dfrac{\mu_a(\lambda_2)\Phi(\lambda_2)}{\mu_a(\lambda_1)\Phi(\lambda_1)}\right] = p_0(\lambda_1, a) \cdot (1 - \alpha_a) \\ P_b = p_0(\lambda_1, b) \cdot \left[1 - \dfrac{\mu_b(\lambda_2)\Phi(\lambda_2)}{\mu_b(\lambda_1)\Phi(\lambda_1)}\right] = p_0(\lambda_1, b) \cdot (1 - \alpha_b) \end{cases}$$

As a consequence, Eq. 4 can be written as,

$$p_0(\lambda_1) - p_0(\lambda_2) = p_0(\lambda_1, a) \cdot (1 - \alpha_a) + p_0(\lambda_1, b) \cdot (1 - \alpha_b) \tag{5}$$

Consequently,by using the notation $\delta p = p_0(\lambda_1) - p_0(\lambda_2)$, we have,

$$\delta p = [p_0(\lambda_1, a) + p_0(\lambda_1, b)] - [p_0(\lambda_2, a) + p_0(\lambda_2, b)] \tag{6}$$

Therefore,

$$\delta p = [p_0(\lambda_1, a) - p_0(\lambda_2, a)] - [p_0(\lambda_1, b) + p_0(\lambda_2, b)] = p_0(\lambda_1, a) \cdot (1 - \alpha_a) + p_0(\lambda_1, b) \cdot (1 - \alpha_b) \tag{7}$$

From the above,

$$p_0(\lambda_2, a) = \alpha_a \cdot p_0(\lambda_1, a) \text{ and, } p_0(\lambda_2, b) = \alpha_b \cdot p_0(\lambda_1, b) \tag{8}$$

Now, if the ratio between the initial pressures of the two chromophores for a given wavelength is calculated,

$$\begin{cases} \dfrac{p_0(\lambda_1, b)}{p_0(\lambda_1, a)} = \dfrac{\Gamma \mu_b(\lambda_1) \Phi(\lambda_1)}{\Gamma \mu_a(\lambda_1) \Phi(\lambda_1)} = \dfrac{\mu_b(\lambda_1)}{\mu_a(\lambda_1)} = m_1 \Rightarrow p_0(\lambda_1, b) = m_1 p_0(\lambda_1, a) \\ \\ \dfrac{p_0(\lambda_2, b)}{p_0(\lambda_2, a)} = \dfrac{\Gamma \mu_b(\lambda_2) \Phi(\lambda_2)}{\Gamma \mu_a(\lambda_2) \Phi(\lambda_2)} = \dfrac{\mu_b(\lambda_2)}{\mu_a(\lambda_2)} = m_2 \Rightarrow p_0(\lambda_2, b) = m_2 p_0(\lambda_2, a) \end{cases}$$

If we replace in Eq. (7) $p_0(\lambda_1, b)$, $p_0(\lambda_2, a)$ and $p_0(\lambda_2, b)$, we obtain,

$$\delta p = p_0(\lambda_1, a) + m_1 p_0(\lambda_1, a) - \alpha_a p_0(\lambda_1, a) - m_2 \alpha_a p_0(\lambda_1, a)$$

then,

$$\delta p = p_0(\lambda_1, a)[1 + m_1 - \alpha_a - m_2 \alpha_a]$$

and,

$$p_0(\lambda_1, a) = \frac{\delta p}{1 + m_1 - \alpha_a - m_2 \alpha_a} = \frac{\delta p}{\zeta} \tag{9}$$

To summarise, the initial pressures created by the two chromophores a and b at λ_1 and λ_2, can be calculated using Eqs. 10

$$\begin{cases} p(\lambda_1, a) &= \dfrac{\delta p}{\zeta} \\ p(\lambda_1, b) &= m_1 \dfrac{\delta p}{\zeta} \\ p(\lambda_2, a) &= \alpha_a \dfrac{\delta p}{\zeta} \\ p(\lambda_2, b) &= m_2 \alpha_a \dfrac{\delta p}{\zeta} \end{cases} \tag{10}$$

In the equations above, m is the ratio between the absorption coefficient of two chromophores at a given wavelength, α is telling us how the initial pressure created by a given chromophore depends on the wavelength, and finally ζ is a dimensionless quantity including both m and α but does not have a specific meaning.

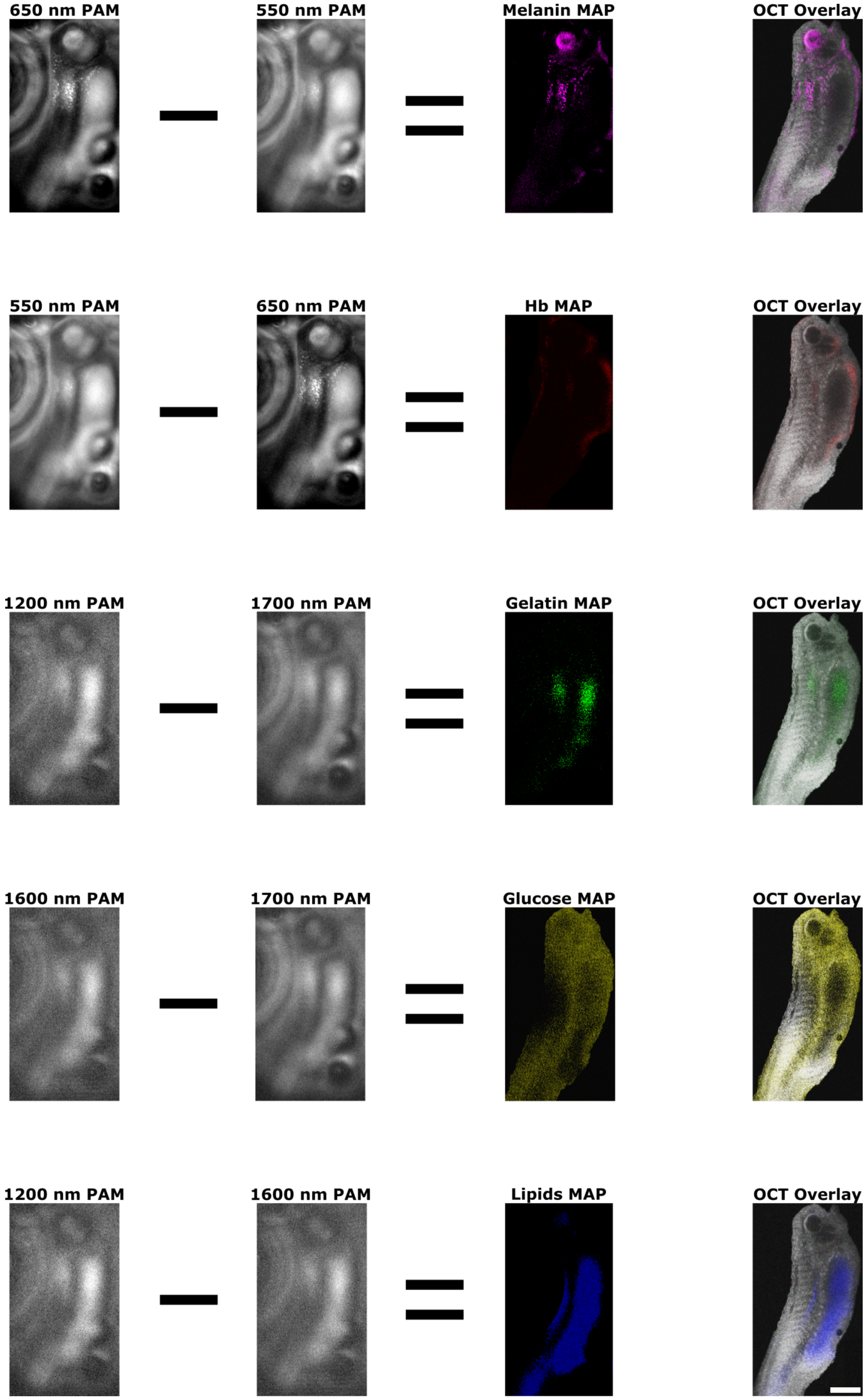

Figure S1. Visual illustration of the mapping technique (theoretically described in Section 1) to produce spatial distribution maps of various chromophores. On the first and second column PAM images obtained by operating OS1 at the wavelength indicated in the labels of the figures are depicted. On the third column, qualitative illustrations of the OS-PAM spatial distribution maps of melanin, Hb, collagen, glucose and lipids in a tadpole at developmental stage 37/38 are presented. On the right column, the OS-PAM images are overlaid over structural OCT images of the same tadpole. PAM imaging artefacts are cancelled out by the technique described in Section 2. The final images obtained on the right column are denoised (using imageJ) and their contrast enhanced by histogram equalization. The scale bar, the same on all figures is 0.5 mm.
